# Randomized clinical trial of streaming dichoptic movies versus patching for treatment of amblyopia in children aged 3 to 7 years

**DOI:** 10.1038/s41598-022-08010-9

**Published:** 2022-03-09

**Authors:** Reed M. Jost, Lindsey A. Hudgins, Lori M. Dao, David R. Stager, Becky Luu, Cynthia L. Beauchamp, Jeffrey S. Hunter, Prashanthi Giridhar, Yi-Zhong Wang, Eileen E. Birch

**Affiliations:** 1 Pediatric Vision Laboratory, Retina Foundation of the Southwest, 9600 North Central Expressway, Suite 200, Dallas, TX 75231 USA; 2 Department of Ophthalmology, University of Texas Southwestern Medical Center, Dallas, TX USA; 3 ABC Eyes – Pediatric Ophthalmology, Dallas, TX USA; 4 Pediatric Ophthalmology & Adult Strabismus, Plano, TX USA; 5 Heaton Eye Associates, Tyler, TX USA

**Keywords:** Refractive errors, Vision disorders, Paediatric research, Paediatric research

## Abstract

Contrast-rebalanced dichoptic movies have been shown to be an effective binocular treatment for amblyopia in the laboratory. Yet, at-home therapy is a more practical approach. In a randomized clinical trial, we compared dichoptic movies, streamed at-home on a handheld 3D-enabled game console, versus patching as amblyopia treatment. Sixty-five amblyopic children (3–7 years; 20/32–125) were randomly assigned to one of two parallel arms, binocular treatment (3 movies/week) or patching (14 h/week). The primary outcome, change in best corrected visual acuity (BCVA) at the 2-week visit was completed by 28 and 30, respectively. After the primary outcome, both groups of children had the option to complete up to 6 weeks of binocular treatment. At the 2-week primary outcome visit, BCVA had improved in the movie (0.07 ± 0.02 logMAR; *p* < .001) and patching (0.06 ± 0.01 logMAR; *p* < 0.001) groups. There was no significant difference between groups (CI_95_%: − 0.02 to 0.04; *p* = .48). Visual acuity improved in both groups with binocular treatment up to 6 weeks (0.15 and 0.18 logMAR improvement, respectively). This novel, at-home, binocular movie treatment improved amblyopic eye BCVA after 2 weeks (similar to patching), with additional improvement up to 6 weeks. Repeated binocular visual experience with contrast-rebalanced binocular movies provides an additional treatment option for amblyopia.

Clincaltrials.gov identifier: NCT03825107 (31/01/2019).

In prospective single-arm studies, contrast-rebalanced dichoptic movies have been used as an effective treatment for children aged 4 to 12 years with amblyopia. With as little as 9 h of viewing time, mean improvement of visual acuity of the amblyopic eyes was 1.5 to 2.0 lines^1,2^. While these laboratory studies provided proof-of-principle data for the treatment approach, at-home treatment is a more practical alternative. More recently, both a single arm study and a randomized clinical trial allowed children to view cloud-based movie content at home using a head-mounted display (a commercially available smartphone in a virtual reality headset) with reduced fellow eye contrast, reporting a mean visual acuity improvement of 1.5 − 1.8 lines within 12 weeks^3,4^. However, head-mounted displays are difficult to use by preschool children because of their size and weight, as well as the visual isolation imposed during wear. Additionally, while there is an objective log time during which movies were streamed, the headset makes it impossible for a parent to periodically check whether the child has their eyes open while the movies are playing.

There is evidence that younger children (≤ 7 years) are more responsive to amblyopia treatments^5,6^, including treatment with contrast-rebalanced dichoptic movies^1^. Here, we explored an alternative approach to at-home amblyopia treatment in younger children with contrast-rebalanced dichoptic movies. In a randomized clinical trial, we evaluated the effectiveness of viewing cloud-based contrast-rebalanced dichoptic movies on a handheld device in children aged 3 to 7 years compared with standard-of-care patching treatment^7^.

## Methods

The research protocol adhered to the tenets of the Declaration of Helsinki, was approved by the University of Texas Southwestern Medical Center Institutional Review Board, and conformed to requirements of the US Health Insurance Portability and Accountability Act of 1996. Written informed consent was obtained from the parent who accompanied their child. The protocol is available on www.clinicaltrials.gov (identifier: NCT03825107 [31/01/2019]). Additionally, this study follows the recommendations proposed by the CONSORT Statement.

### Participants

Children aged 3 to 7 years who were diagnosed by pediatric eye specialists as having amblyopia due to strabismus, anisometropia, or both (combined mechanism) were eligible. Inclusion criteria were: (1) amblyopic eye visual acuity of 0.2 to 0.8 logMAR; (2) fellow eye visual acuity of − 0.1 to 0.2 logMAR; (3) interocular visual acuity difference of ≥ 0.2 logMAR; (4) anisometropia or corrected strabismus (< 5pd); (5) wearing glasses for at least 8 weeks with no change in visual acuity over two visits; and (6) must be able to see the full movie screen (both amblyopic and fellow eye components) with fellow eye contrast set to 20%. Exclusion criteria were: (1) prematurity of 8 weeks or more; (2) coexisting ocular or systemic disease; (3) developmental delay; and (4) significant ocular misalignment (≥ 5 pd). Diagnoses, alignment, prior treatment, and visual acuity for visits prior to enrollment were extracted from the referring doctor’s medical records.

### Study design

**Figure 1:**
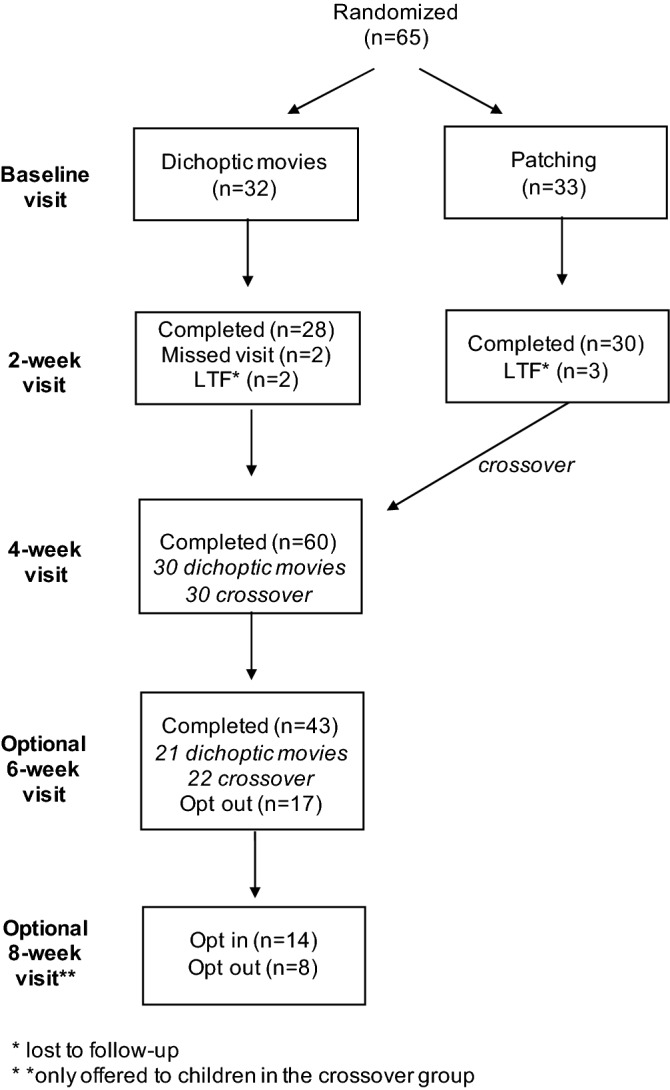
CONSORT diagram.

This study was a randomized clinical trial (parallel group design). At the baseline visit, eligibility was ascertained and vision assessments were conducted. Children were randomly assigned to watch contrast-rebalanced dichoptic animated movies (experimental treatment) or to patch the fellow eye 2 h/day every day (standard-of-care amblyopia treatment) for 2 weeks. The 2-week time point was chosen for the primary outcome based on our pilot studies of contrast-rebalanced dichoptic movies^1,2^ and our randomized treatment trial of contrast-rebalanced dichoptic games^8^. Randomization was performed through the website www.randomization.org with a coded distribution of a 1:1 ratio, with blocks of 12 allocations. Investigators had access to the randomization assignment, which was sealed in a numbered envelope, only after the child was enrolled. At the 2-week primary outcome visit, children in the patching group crossed over to the dichoptic animated movie group and all participants were asked to return for a secondary outcome visit vision assessment at 4 weeks. Families had the option to continue with the dichoptic movies for a total of 6 weeks (Fig. 1). All children returned for a secondary outcome visit at 4 weeks. After that, children in the dichoptic movie group had the option to continue an additional 2 weeks and children who were initially in the patching group had the option to continue with movies for an additional 4 weeks (up to 6 weeks of movies for both groups). Children who continued had follow-up visits with vision assessments every 2 weeks (14 ± 3 days).

### Dichoptic animated movies

Eighteen popular animated feature films were modified using custom MATLAB software to allow dichoptic presentation on a New Nintendo 3DS XL platform (Nintendo Corporation, Kyoto, Japan), which was loaned to the child for the duration of the study so that movies could be watched at home. This Nintendo device allowed for dichoptic viewing without the need for anaglyphic, polarized, or shutter glasses or a VR headset. The movies had the same design as in our recent prospective study of contrast-rebalanced movies in which children came into the laboratory to watch movies on a passive 3D display^1,2^. Briefly, odd lines in the Nintendo 3D display were visible to one eye, and even lines were visible to the other eye. A patterned image mask composed of irregularly shaped blobs was multiplied with the images seen by the amblyopic eye, and the inverse patterned mask was multiplied with the images seen by the fellow eye, so that different parts of the display were seen by each eye. Blobs of the movie seen by the amblyopic eye always had high contrast (100%), whereas the complementary blobs seen by the fellow eye had reduced contrast. Because the blobs had Gaussian edges, the edges of the blobs overlapped and were seen by both eyes with differing contrasts, preventing any significant rivalry. The shape and location of the blobs were varied dynamically every 10 s. Fellow eye contrast started at 20% for the first movie and incremented by 10% for each subsequent movie (i.e., 20%, 22%, 24%, 26%, 29%, …). Movies were uploaded to our Amazon Web Services (AWS; Seattle, WA, USA) streaming service in two-week packets, each containing 6 movies that preserved the order of fellow eye contrasts from low to high.

### Study protocol

Children assigned to the dichoptic movie arm were asked to watch 3 movies per week (about 4.5 h per week) during each 2-week period, based on the 4–5 h per week used in our previous contrast rebalanced binocular treatment studies and randomized treatment trials^1,2,8–10^. During their visit, we provided a link on their loaned Nintendo for the set of 6 movies that should be watched during the next two weeks. Movie names were numbered on the Nintendo device, in order by fellow eye contrast, and the family was instructed that the movies should be viewed in that order at home with the date recorded on a paper log sheet. The log sheet had pictures of the title frames from each of the 6 movies with a space next to each for the parent to write in the date it was watched. Fellow eye contrast of movies 1–6 incremented from 20 to 32% during the first 2 weeks, movies 7–12 incremented from 35 to 57% during weeks 3 and 4 and, finally, movies 13–18 incremented from 63 to 100% if children participated in the optional weeks 5 and 6. Children assigned to the patching arm were supplied with patches and asked to patch two hours per day for two weeks. At the 2-week visit, children were loaned a Nintendo and began watching movies, starting with 20% fellow eye contrast for the next two weeks, and had the option of completing up to 6 weeks of movies.

### Vision assessments

Crowded monocular best-corrected visual acuity (BCVA) was tested using the Amblyopia Treatment Study HOTV protocol for 3- to 6-year olds (N = 37)^11–13^ and the E-ETDRS protocol for 7-year olds (N = 16)^14–16^. A few of the youngest children who were unable to complete ATS-HOTV were tested with Allen Pictures on the Mentor Baylor Visual Acuity Tester II (BVAT-II; Mentor Ophthalmics, Inc., Norwell, MA, USA; N = 7). Whichever visual acuity test was used for baseline assessment was also used at all follow-up visits. Stereoacuity was assessed with the Randot Preschool Stereoacuity Test and the Stereo Butterfly Test [Stereo Optical Inc, Chicago, IL]^17^; stereoacuity was converted to log arcsec for analyses; nil stereoacuity was arbitrarily assigned a value of 4.0. Extent of suppression was assessed using the Worth-4 dot test at 7 distances (3 m, 2 m, 1 m, 0.67 m, 0.5 m, 0.33 m, and 0.16 m)^18^; the farthest distance at which the child reported 4 dots was converted into the area of suppression scotoma in log degrees. Depth of suppression using a dichoptic eye chart that identifies the non-preferred eye/preferred eye contrast ratio (i.e., balance point) at which the child can overcome suppression and report letters presented to each eye with equal likelihood (Contrast Balance Index; CBI)^19,20^.

### Sample size and statistical analysis

The primary outcome was change in amblyopic eye BCVA at the 2-week visit. The analysis was conducted with a modified intent-to-treat approach, limited to participants who completed the 2-week visit within the pre-specified analysis window (± 3 days after the baseline/randomization visit) and no imputation for missing data. A t-test was used to compare the visual acuity change in the two treatment groups. Based on our recent prospective study of contrast re-balanced dichoptic movies as a treatment for amblyopia^1,2^, we anticipated a 0.15 ± 0.10 logMAR (7.5 letters) improvement in visual acuity at the 2-week primary outcome visit for children assigned to the dichoptic movie arm. For children assigned to the patching group, we anticipated a 0.07 ± 0.10 logMAR (3.5 letters) improvement at 2 weeks based on results from our prior study of patching versus a contrast-rebalanced dichoptic game as a treatment for amblyopia^8^. With these expected means, a sample size of 28 per group (56 total) would provide 85% power to declare that the two groups have significantly different means, using a two-sided *p*-value of less than 0.05^21^. We enrolled and randomized 65 children (32–33 per group) to account for potential loss to follow-up.

As a secondary analysis of BCVA at the 2-week primary outcome visit, one sample t-tests were conducted to determine whether amblyopic eye BCVA improvement at the 2-week visit was significant in each group. In addition, confidence intervals (95%) were calculated to determine whether there were significant improvements in stereoacuity, depth of suppression, or extent of suppression at the 2-week primary outcome for each group. Exploratory analyses were conducted to determine whether the improvement in visual acuity at the 2-week visit was associated with the child’s baseline visual acuity, baseline stereoacuity, or their response to conventional treatment with glasses and patching prior to enrollment. Response to conventional treatment was categorized as “responded to treatment” if visual acuity improvement ≥ 0.1 logMAR occurred with glasses and patching over two or more visits prior to enrollment. Children were classified as having had “no prior response” to conventional treatment if there was no improvement in visual acuity over two visits separated by at least 8 weeks.

## Results

Between Jan 31, 2019 and May 25, 2021, 65 eligible children were enrolled. Of the 65 participants, 5 left the study prior to the 2-week primary outcome visit (2 in the movies and 2 in the patching group did not return and were lost to follow-up; 1 child in the patching group withdrew from the study due to an unrelated illness) and 60 completed the study through the 4-week visit (Fig. 1). Baseline characteristics are listed in Table 1.

**Table 1:** Baseline characteristics.

	Contrast-rebalanced movies (N = 30 )	Patching (N = 30 )
Female, n (%)	15 (50%)	19 (63%)
**Age, years, n**		
3	3	5
4	5	3
5	3	3
6	11	11
7	8	8
Mean ± SD, years	6.0 ± 1.4	6.1 ± 1.5
**Prior amblyopia treatment**, **n**		
None	6	7
Patching	18	19
Patching + atropine	4	3
Patching + binocular treatment	1	0
Patching + atropine + binocular treatment	1	1
**Weeks in glasses, n**		
8–13	3	3
> 13–26	3	4
> 26	24	23
**Cause of amblyopia, n**		
Strabismus	8	11
Anisometropia	14	15
Combined	8	4
**AE BCVA, logMAR, n**		
0.2–0.6 (20/30–80)	26	25
0.7–0.8 (20/100–125)	4	5
Mean ± SD, logMAR	0.46 ± 0.17	0.44 ± 0.20
**FE BCVA, logMAR, n**		
− 0.1 (20/16)	3	8
0.0 (20/20)	13	11
0.1 (20/25)	8	10
0.2 (20/30)	6	1
Mean ± SD, logMAR	0.06 ± 0.09	0.01 ± 0.09

### Adherence to protocol

For the first 2 weeks, children randomized to the movie group watched 5.7 ± 0.7 movies (approximately 8.6 h; 95% adherence) and children in the patching group averaged 30.0 ± 11.0 h of patching (107% adherence). In subsequent weeks, the movie group watched 6.4 ± 1.1 movies (107% adherence) between the 2-week and 4-week visits and 5.7 ± 1.3 movies (95% adherence) between the 4-week and 6-week visits. Children in the patching group crossed over to the movie group after the 2-week visit and watched 5.4 ± 1.3 movies (90% adherence) between the 2-week and 4-week visits, 5.3 ± 1.5 movies (88% adherence) between the 4-week and 6-week visits, and 5.8 ± 0.6 movies (97% adherence) between the 6-week and 8-week visits. The dated, parental logs of movies watched by date obtained at each visit indicated that treatments were administered as intended, with fellow eye contrast incrementing throughout study period.

### Visual acuity

**Figure 2:**
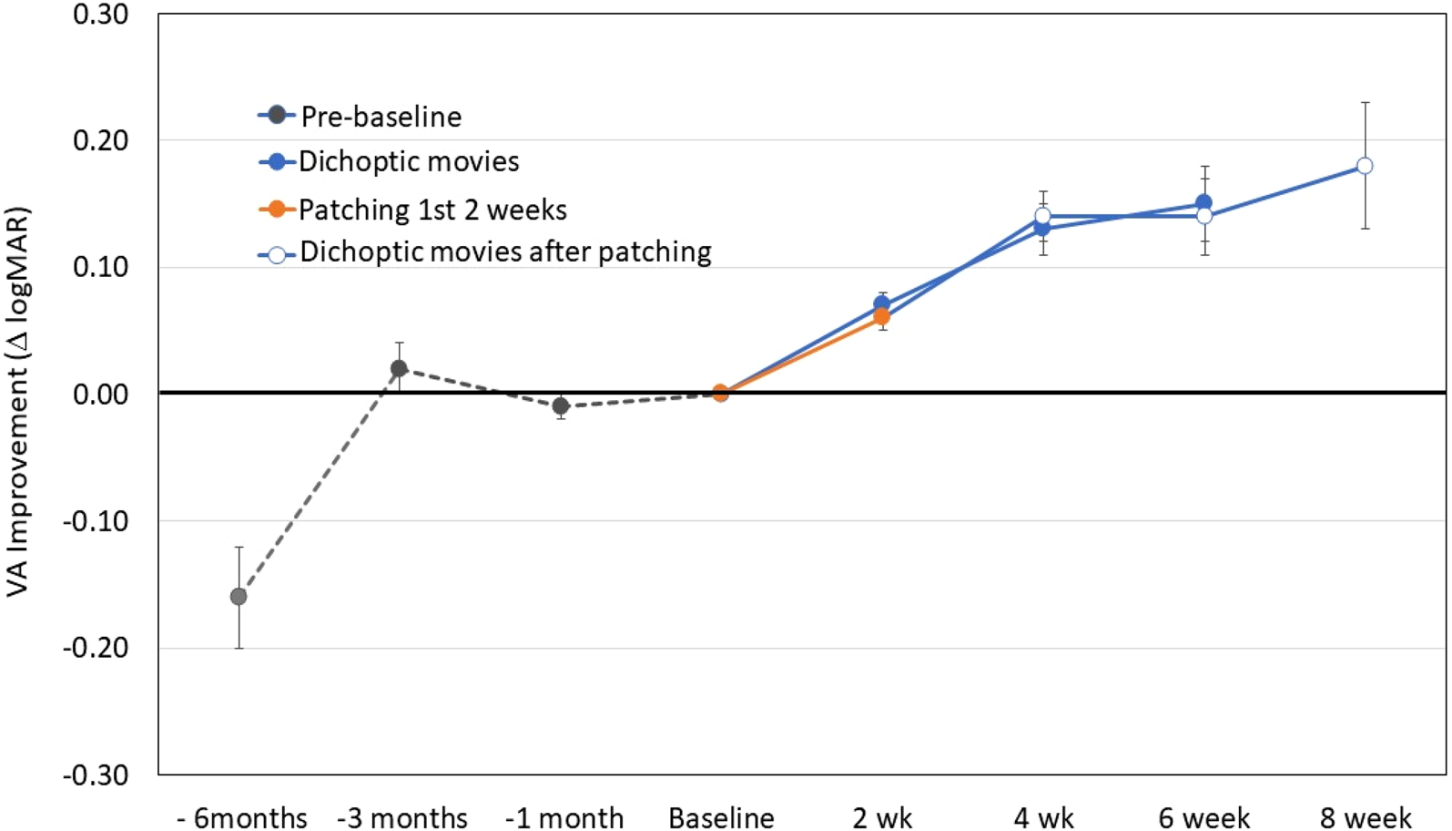
Amblyopic eye visual acuity improvement relative to baseline for 60 children randomized to watch contrast rebalanced dichoptic movies or patching for 2 weeks. Also shown are visual acuity differences from baseline extracted from medical records at 1, 3, and 6 months prior to the baseline/randomization visit and further visual acuity improvement with continued viewing of dichoptic movies by children in the dichoptic movie group at 4 and 6 weeks and by children who crossed over to watch dichoptic movies after their initial two weeks of patching treatment and participated through 8 weeks.

Visual acuity measurements for the amblyopic eye acquired by the referring pediatric ophthalmologists at 1 month, 3 months, and 6 months prior to the study baseline visit were identified in the medical records and means ± SDs are plotted in Fig. 2. Not all participants attended clinical visits at all time points. Some children were referred within 3 months of their initial diagnosis and a few children were not able to participate in visual acuity testing at clinical visits due to their young age prior to being referred to the study. Overall, we had visual acuity data from 87% at 1 month prior, 68% at 3 months prior, and 55% at 6 months prior to baseline. For children who had visual acuity test results at 3 and 6 months prior to enrollment, a mean improvement in visual acuity with treatment by spectacles or spectacles plus patching of 0.18 logMAR (just under 2 lines) was observed. However, despite continuing treatment, there was little change in visual acuity between 3 months and 1 month prior to enrollment; i.e., visual acuity was stable at the time of enrollment.

At the 2-week primary outcome visit, similar improvement in amblyopic eye best-corrected visual acuity was found in the movie and patching groups (0.07 vs. 0.06 logMAR; CI_95_% for the difference between groups: − 0.02 to 0.04; t_56_ = 0.70, *p* = 0.48; Fig. 2). Treatment with either movies or patching resulted in significant improvement in visual acuity [0.07 ± 0.05 logMAR (t_27_ = 7.00, *p* < 0.0001; Cohen’s d = 1.4) and 0.06 ± 0.05 logMAR (t_29_ = 8.00, *p* < 0.0001; Cohen’s d = 1.2), respectively]. Within the movie group, 13 children (46%, CI_95_%: 30–64%) improved by 0.10 logMAR or more and 8 (29%, CI_95_%: 15–47%) improved by 0.20 or more; 54% did not improve. In the patching group, 14 children (47%, CI_95_%: 30–64%) improved by 0.10 logMAR or more and 5 (17%, CI_95_%: 7–34%) improved by 0.20 logMAR; 53% did not improve. At the 2-week visit, 4 children (14%; CI_95_%:, 6–31%) in the movie group and 3 children (10%; CI_95_%: 3–26%) of children in the patching group had ≤ 0.1 logMAR interocular difference in visual acuity (recovered).

Visual acuity continued to improve in the movie group after the 2-week primary outcome visit, with gains of 0.13 ± 0.11 logMAR by 4 weeks and 0.15 ± 0.10 logMAR by 6 weeks (Fig. 2). The patching group showed similar gains after crossing over to movies at 2 weeks; by week 8, they gained 0.18 ± 0.07 logMAR (Fig. 2).

The option to continue the movie treatment past the 4-week visit for up to 6 weeks of the movie treatment was elected by 35 (58%) children. Among the 25 families who did not elect to continue, there were multiple reasons: achievement of normal visual acuity (n = 4), compliance difficulty (n = 7), no improvement of visual acuity (n = 9), lab closure due to COVID-19 (n = 2), and unknown reasons (n = 3). After 6 weeks of watching contrast re-balanced dichoptic movies (6-week visit for the movie group and 8-week visit for the patching group) 26% (CI_95_%: 14–42%) of children had ≤ 0.1 logMAR (≤ 1 line) interocular difference in visual acuity (recovered).

### Exploratory analyses

#### Stereoacuity and suppression

At the 2-week primary outcome visit, stereoacuity was improved in the movie group (0.12 log arcsec; CI_95_%: 0.02–0.22) and this improvement was maintained through the 4-week visit (0.11 log arcsec; CI_95_%: 0.04–0.18). The patching group did not have improved stereoacuity at either the 2-week (0.02 log arcsec; CI_95_%: − 0.16–0.20) or 4-week visit (-0.03 log arcsec; CI_95_%: − 0.15–0.09). Neither depth of suppression nor extent of suppression significantly improved in either group at the 2- week or 4-week visit.

#### Baseline factors

**Figure 3:**
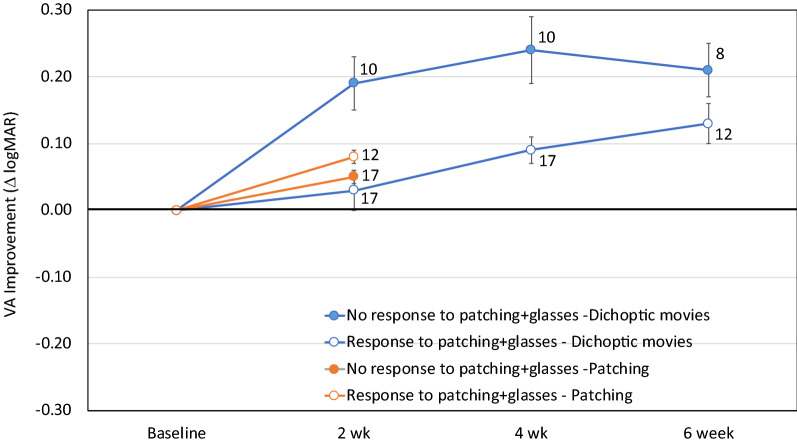
Visual acuity improvement in the movie and patching groups in children who had a clinical history of prior visual acuity improvement when treated with glasses and patching and children who had no visual acuity improvement when treated with glasses and patching. Numbers to the right of each data point indicate the n at each timepoint for each subgroup. Note that 3 children in the movie group and 1 child in the patching group were too young to provide visual acuity data on two visits prior to the enrollment visit and, as a result, could not be categorized as no response or response. These children were excluded from this analysis.

Within the movie group, there was a trend for children with poorer baseline visual acuity ≥ 0.4 logMAR) to have more visual acuity improvement at the 2-week primary outcome visit than children with better baseline visual acuity (≤ 0.3 logMAR; t_26_ = 1.99, *p* = 0.06). This trend was not apparent in the patching group (t_28_ = 1.99, *p* = 0.19). There was not a significant association between baseline stereoacuity (nil vs ≤ 3.3 log arcsec) and visual acuity improvement at the 2-week primary outcome visit in either the movie or patching group (t_26_ = 0.91, *p* = 0.37 and t_28_ = 0.90, *p* = 0.38, respectively). Within the movie group, children who had a clinical history of no visual acuity improvement in response to treatment with glasses and patching had a larger visual acuity improvement at the 2-week primary outcome visit than children who previously had responded to treatment with glasses and patching (0.19 ± 0.04 logMAR vs 0.03 ± 0.03 logMAR; t_25_ = 3.01, *p* = 0.006; Fig. 3). There was no significant difference between the baseline visual acuities of these two subgroups (0.47 ± 0.16 logMAR for the group that had no response and 0.45 ± 0.17 logMAR for the subgroup that responded to glasses and patching; t_25_ = 0.08, *p* = 0.94). This difference remained at the 4-week visit (*p* = 0.002) but not at the 6-week treatment (*p* = 0.16, Fig. 3). There was not a significant association between age at enrollment (3–5 years vs 6–7 years) and visual acuity improvement at the 2-week primary outcome visit in either the movie or patching group (t_27_ = 0.20, *p* = 0.84 and t_29_ = 0.99, *p* = 0.33, respectively).

## Discussion

Both the contrast-rebalanced dichoptic movie and patching groups had clinically significant improvement in amblyopic eye BCVA at the 2-week primary outcome visit, with a similar amount of improvement in each group. In these young children, contrast-rebalanced dichoptic movies are as effective as patching. Therefore, dichoptic movies could offer a break from patching without stalling progress or risking recurrence, and thus provide a second option for children who are or have become noncompliant with patching. The mean BCVA improvement at the 2-week visit was modest at 0.07 logMAR, but continued visual acuity improvement was observed with use of contrast-rebalanced dichoptic movies for 6 weeks, for a mean improvement of 0.15 − 0.18 logMAR. After 6 weeks of viewing contrast-rebalanced dichoptic movies at home, 29% of children in the dichoptic movie group had ≤ 0.1 logMAR (≤ 1 line) interocular difference in visual acuity (recovered). These results are similar to those reported in a recent randomized trial of 4- to 7-year-old using a head-mounted display to view dichoptic movie content at-home as a treatment for amblyopia^4^. Additionally, a modest improvement in stereoacuity was observed in the dichoptic movie group at the primary outcome and 4-week visit. Reports of stereoacuity improvements with other dichoptic therapies for amblyopia in children are variable, with some reporting improvement^22–25^ and others reporting no change^4,26,27^. The factors that contribute to this variability in stereoacuity outcomes of binocular treatment remain to be determined.

In the movie group, but not in the patching group, children who had a clinical history of no visual acuity improvement in response to treatment with glasses and patching had a larger visual acuity improvement at the 2-week primary outcome visit than children who previously had responded to treatment with glasses and patching. This could reflect better adherence to binocular treatment in the setting of a randomized clinical trial than adherence to patching prior to enrollment or, alternatively, may result from individual differences in treatment efficacy of binocular versus patching treatments^28^.

Less BCVA improvement was seen with this at-home contrast-rebalanced dichoptic movie treatment than we previously reported for supervised use of the same movies in-lab^1^. This may have been a consequence of broader inclusion criterion, which included children with baseline amblyopic eye visual acuity of 0.2 logMAR. Including milder amblyopia in the at-home study may have allowed less room for visual acuity improvement than in the in-lab study. The at-home study also included a much smaller proportion of children with baseline amblyopic eye visual acuity of > 0.7 logMAR than the in-lab study (12% vs 30%), further limiting the maximum amount of improvement that could be observed. Other factors that may have contributed to differences between the two studies include attention to the movies in-lab compared to at-home and the better quality of the larger passive LG Electronics (Englewood, NJ, USA) monitor used in-lab compared to the image of the handheld Nintendo device used for at-home viewing.

Because children had stable baseline BCVA for the 1-month period prior to enrollment and randomization even though most were patching, it was surprising to observe BCVA improvement in the group randomized to patching at the primary outcome visit. It is possible that patching adherence was poor just prior to enrollment and may have improved as a result of participation in a randomized clinical trial^29^. Without objective compliance data, it is not possible to attribute the response to patching solely to an improvement in treatment adherence^28,30^.

Potential advantages of the contrast re-balanced dichoptic movies used here are at-home therapy, movie content appropriate for 3- to 7-year-old children, requirement for only 4.5 h per week of treatment time, no need for a headset or polarizing glasses, and excellent adherence. Potential disadvantages are increased screen time relative to patching, need for a passive 3D screen and access to wi-fi, and the requirement to focus solely on the movie (patching can be accomplished while the child is engaged in other activities).

Strengths of the study include randomization, spectacle adaptation period of ≥ 8 weeks prior to enrollment, no requirement for polarizing glasses or a headset to view dichoptic movies, and engaging movies that supported excellent adherence. In addition, while contrast re-balanced games have been shown to be effective as an amblyopia treatment for children aged 4 years and older^8,23,26,31^, the games are somewhat complex and can be challenging for children aged < 6 years. Dichoptic movies and videos, then, may provide an advantage over games in ease of use for the youngest children who may benefit from early treatment of amblyopia^5,6^. Use of a 3D display to separate images for each eye, rather than a head-mounted VR headset, may provide advantages for young children who find the headsets uncomfortable due to their size and weight size and weight, and may dislike the visual isolation imposed during wear. Additionally, headset wear makes it impossible for parents to watch the movie with their child or even check whether their child has their eyes open while the movies are playing.

Limitations of the study include the short duration of randomly assigned treatment (2 weeks), followed by a crossover. Nonetheless, groups had continuing gains in BCVA throughout their 6–8 weeks in the study and the gains in BCVA were comparable to those reported for young children in randomized clinical trials of patching and binocular games that included a spectacle adaptation period of ≥ 8 weeks^27,32–34^. Another limitation of the study is the use of three different visual acuity tests. While most participants were tested using the ATS-HOTV protocol, 7 of the youngest participants were tested with Allen pictures, which may overestimate visual acuity compared to letter optotypes^35^, and 16 of the oldest participants were tested with the E-ETDRS protocol, which may have a slight bias toward worse performance of amblyopic eyes than ATS-HOTV in children^36^. Regardless, the same visual acuity test was repeated at each child’s visit, allowing determination of changes in BCVA relative to baseline. A limitation of presenting contrast-rebalanced dichoptic movies on a handheld device is that any particular hardware may become obsolete in a few years. Nonetheless, there are many potential devices for presenting dichoptic movies. For this study we chose to use an inexpensive device familiar to children, the New Nintendo 3DS XL, but the same approach could be easily transferred to other hardware platforms. Another limitation is the lack of objective monitoring of adherence for both patching and the movie treatment, instead, relying on parental written logs in this study. This limitation is typical of randomized clinical trials designed to evaluate the effectiveness of prescribed treatments rather than their efficacy^33,37–41^.

Overall, this novel, at-home, binocular movie treatment was effective in improving amblyopic eye BCVA, with additional improvements observed with up to 6 weeks of treatment. Repeated binocular visual experience with contrast-rebalanced dichoptic movies provides an additional treatment option for amblyopia.
